# Long‐Acting Cabotegravir/Rilpivirine Reduces Immune‐Activation and ‐Senescence in People With HIV With CMV Co‐Infection

**DOI:** 10.1111/imm.70154

**Published:** 2026-06-16

**Authors:** Mariasilvia Guardiani, Eeva Tortellini, Anna Carraro, Maria Antonella Zingaropoli, Lorenzo Ansaldo, Alessandra Grimaldi, Serena Vita, Federica Dominelli, Fabio Mengoni, Caterina Pasquazzi, Ombretta Turriziani, Maria Rosa Ciardi, Vincenzo Vullo, Claudio Maria Mastroianni, Raffaella Marocco, Cosmo Del Borgo, Miriam Lichtner

**Affiliations:** ^1^ Department of Public Health and Infectious Diseases Sapienza University of Rome Rome Italy; ^2^ Department of Neurosciences, Mental Health, and Sense Organs, NESMOS Sapienza University of Rome Rome Italy; ^3^ Infectious Diseases Unit, SM Goretti Hospital Sapienza University of Rome Latina Italy; ^4^ Department of Molecular Medicine Sapienza University of Rome Rome Italy

**Keywords:** CAB/RPV‐LA, CMV, flow cytometry, HIV, immune‐activation and immune‐senescence, PWH

## Abstract

Despite the effectiveness of combined antiretroviral therapy (ART), HIV infection remains a chronic condition characterised by persistent inflammation and immune activation, likely associated with viral persistence and other factors such as cytomegalovirus (CMV) co‐infection. Long‐acting (LA) injectable formulations, a newer class of ART with improved and sustained bioavailability, may help modulate the HIV‐associated immunoinflammatory state. Therefore, we analysed dynamic changes in lymphoid immune‐activation and ‐senescence markers in people with HIV (PWH) switching to LA injectable cabotegravir and rilpivirine (CAB/RPV‐LA), compared with PWH who continued oral ART by choice. The relationship between CMV‐specific immune responses and phenotypic T‐cell alterations was also evaluated. T‐cell immune‐activation (CD38 + HLA‐DR+) and ‐senescence (CD28‐CD57+) were measured at baseline (T0), at 4 (T4), 28 (T28), 48 (T48) and at 72 (T72) weeks after switching to CAB/RPV‐LA. A control group (CG) of PWH (PWH‐CG) on daily oral ART was studied at T0 and at 48 weeks (T48). Furthermore, CMV‐specific cellular and humoral immune responses were assessed. Thirty‐seven PWH switching to CAB/RPV‐LA and nine PWH‐CG were enrolled. In PWH‐LA, total CD4 levels remained stable over time, while CD8 levels decreased significantly at T72 versus T0 and T4. In CG, an increasing CD4 and decreasing CD8 trend was observed. The CD4/CD8 ratio remained stable in both PWH‐LA and ‐CG and showed a nonsignificant increase at T48 in PWH‐CG. Immune‐activation decreased in PWH‐LA: CD4 activation levels were significantly lower at T48 and T72 versus earlier time points and CD8 activation was reduced at T72 versus T0 and T4. Immune‐senescence declined in both compartments, with a significant reduction in CD8 senescence at T28, T48 and T72 compared to earlier time‐points. Delta analysis confirmed a greater reduction in immune‐activation and ‐senescence in PWH‐LA compared to PWH‐CG. Regarding CMV immune response, anti‐CMV antibody levels remained stable with only a minor decline over time, whereas CMV‐specific T‐cell responses transiently increased at T28 before returning to baseline at T48 and T72. Switching to CAB/RPV‐LA is associated with reduced T‐cell activation and senescence, particularly in CD8 T‐cell, suggesting improved immune homeostasis beyond viral suppression alone. However, despite these promising immunological changes, CMV coinfection remains a key driver of residual immune dysfunction.

## Graphical abstract

This study investigates the impact of switching to long‐acting (LA) injectable cabotegravir and rilpivirine (CAB/RPV‐LA) on immune‐activation and ‐senescence in people with HIV (PWH). Thirty‐seven PWH switching to CAB/RPV‐LA and nine continuing oral ART were analysed over 72 weeks. LA therapy led to significant reductions in activation T and senescence T‐cell, especially in CD8 cells, indicating improved immune homeostasis beyond viral suppression. CMV‐specific responses showed transient fluctuations, while antibody levels remained stable. Overall, CAB/RPV‐LA appears to attenuate chronic immune activation, although CMV coinfection continues to influence residual immune dysfunction.

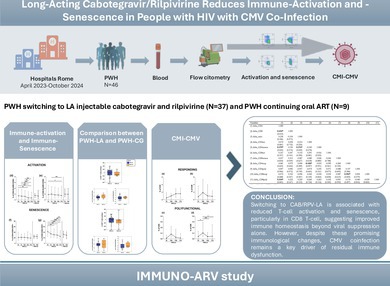

## 1 Introduction

The introduction of Long Acting (LA) injectable drugs in the context of HIV infection represents one of the major pharmacological innovations of the last 20 years and constitutes a new paradigm in the administration of therapy [1, 2]. The first two available drugs of this new category, Cabotegravir/Rilpivirine (CAB/RPV)‐LA, have demonstrated noninferiority in maintaining virological suppression, compared with oral therapy [2, 3, 4, 5, 6]. Furthermore, switching from a daily pill to slow‐release intramuscular combinations constitutes a response to crucial challenges in treatment optimisation, improving adherence, satisfaction and reducing HIV‐related stigma among people with HIV (PWH) [4, 7, 8, 9].

Although the introduction of antiretroviral therapy (ART) has improved the life expectancy of PWH, it fails to completely reverse the effects of viral infection on the immune system [10, 11, 12, 13]. Residual inflammation and chronic immune‐activation persist despite effective viral suppression, contributing to the development of non‐AIDS‐related comorbidities such as cardiovascular disease, renal impairment, bone disorders, cancer and neurocognitive decline [14].

In parallel, chronic HIV infection is associated with immune‐senescence, characterised by the accumulation of highly differentiated T‐cells with reduced proliferative capacity and altered effector functions, commonly identified by the loss of CD28 expression and the acquisition of CD57 [15]. This phenotype is strongly linked to aging‐related comorbidities and further increases the risk of non‐AIDS conditions, even in ART‐treated individuals [16]. These alterations reflect a complex and multifactorial process involving persistent immune stimulation and coinfections [17, 18, 19, 20].

Among these, cytomegalovirus (CMV) co‐infection plays a major role in sustaining immune dysfunctions in PWH [21]. As a persistent infection, CMV drives chronic antigenic stimulation, promoting the expansion of highly differentiated and senescent T‐cell subsets, particularly within the CD8 compartment [22, 23]. Importantly, CMV‐related immune alterations persist even in individuals with long‐term viral suppression and preserved CD4 T‐cell counts, suggesting its contribution to residual immune dysregulation beyond HIV replication alone [24].

Normally, early initiation of ART leads to a decrease in T‐cell activation, particularly for CD8 T‐cells, though full reversal to normal levels is rarely achieved, and changes in the innate immune compartment are more variable [25, 26]. To date, no data are yet available on the modifications on lymphoid immune‐activation and senescence markers following initiation of LA injectable ART. Understanding these changes is essential to evaluate immune restoration beyond viral control.

We aimed to characterise longitudinal changes in immune‐activation and ‐senescence after switching to CAB/RPV‐LA, and to explore the influence of CMV co‐infection on these immune parameters.

## 2 Methods

### 2.1 Study Design and Population

Persistently aviremic PWH, under routine follow‐up at the Infectious Diseases Unit of the SM Goretti Hospital, Latina, Sapienza University, who accepted to switch from a daily oral regimen to a CAB/RPV injectable regimen, following actual guidelines indications [27, 28, 29, 30] were asked to be enrolled in the study (IMMUNO‐ARV 57.23).

As Control Group (CG), PWH with similar clinical characteristics who preferred to maintain their oral daily ART were asked to be included in the study.

During routine clinical testing, peripheral whole blood samples were collected at the following time‐points: before starting CAB/RPV‐LA (T0), after 4 (T4), 28 (T28), 48 (T48) and 72 (T72) weeks, along with demographic, epidemiologic, clinical and laboratory characteristics of all participants. A total of 9 PWH were enrolled in the CG.

Parameters such as body weight, CDC stage, time elapsed from diagnosis, comorbidities, HIV RNA, Hepatitis B core antibody (HBcAb), HBsAg (Hepatitis B virus surface antigen), anti‐HCV antibody (Hepatitis C Virus), kidney and liver function, lipid profile, CD4 nadir, CD4 and CD8 T‐cells count, ratio CD4/CD8, IgG anti‐CMV antibody and CMV‐DNA were collected.

Exclusion for the study were coinfection with HBV (Hepatitis B virus), not complete HIV control (HIV‐RNA undetectable < 12 months), resistance to Integrase Strand Transfer Inhibitors (INSTIs) and/or Nonnucleoside Reverse Transcriptase Inhibitors (NNRTIs) and age < 18 years. Given the relatively small size of the CG compared with the PWH‐LA group, between‐group comparisons should be interpreted with caution, as the study may be underpowered to detect small differences.

### 2.2 T‐Lymphocytes Activation and Immune‐Senescence

#### 2.2.1 Definition of Activation and Senescence Markers

Immune‐activation and ‐senescence of peripheral blood T‐lymphocytes were evaluated in ethylenediaminetetraacetic acid (EDTA) whole blood cells according to a lyse‐and‐wash protocol [31, 32]. On collected blood samples, the percentages of T‐cell (CD4 and CD8) were evaluated.

Since co‐expression of CD38 and HLA‐DR is the key phenotype of the activation of CD4 and CD8 T‐cells, co‐expression of CD38 and HLA‐DR was analysed (CD38 + HLA‐DR+). The immune‐senescent T‐cell phenotype was evaluated as a lack of CD28 expression and expression of the senescence marker, CD57 (CD28‐CD57+) [33].

#### 2.2.2 Flow Cytometry Staining and Acquisition

Briefly, 50 μL of whole blood sample was stained with the following mix of monoclonal antibodies: Pacific, Blue‐conjugated anti‐CD3, FITC‐conjugated anti‐CD28, PE‐conjugated anti‐CD57, PerCp/Cy5.5‐conjugated anti‐HLA‐DR, Pe/Cy7‐ conjugated anti‐CD8, CD38‐ conjugated anti‐APC and APC/Cy7‐ conjugated anti‐CD4 [31]. All antibodies were purchased from Biolegend (BioLegend, San Diego, CA, USA) and used according to the manufacturer's instructions. After an incubation in darkness at 4°C for 20 min, red blood cells were lysed at room temperature for 10 min using the BD lyse solution (BD Biosciences). The cells were then washed in phosphate‐buffered saline (PBS) containing 1% of Foetal Calf Serum (FCS) and fixed in PBS containing 0.5% of formaldehyde (Sigma‐Aldrich) before analysis [31].

The stained samples were acquired using the MACSQuant Flow Cytometer (Miltenyi Biotec, Germany) and analysed using FlowJoTM v10.6.2 software.

### 2.3 Evaluation of CMV‐Specific Responses

#### 2.3.1 Humoral Response (Anti‐CMV IgG)

Serum anti‐CMV IgG titers were determined using a chemiluminescence immunoassay (CLIA) (DiaSorin S.p. A, Saluggia, Italy). The levels of anti‐CMV IgG antibodies were expressed in U/mL (Unit/mL). A positive serologic response was defined as detectable IgG antibodies against CMV over the cut‐off value of > 180 U/mL. The lower detection limit of the assay was < 5 U/mL.

### 2.4 CMV‐Specific T‐Cell Functional Assay

CMV‐specific T‐cell responses were assessed by using an intracellular cytokine cytometric analysis, as previously described [34, 35]. To evaluate CMV‐specific T‐cell functionality, intracellular production of IFNγ, TNFα and IL‐2 was measured, as these cytokines are widely used to characterise antigen‐specific T‐cell responses and to define T‐cell polyfunctionality, a key feature of effective antiviral immunity. ‘Responding T cells’ were defined as those producing any of IFNγ, IL‐2 or TNFα, while ‘polyfunctional T cells’ were defined as those simultaneously producing all three cytokines.

A pool of 15‐mer peptides with 11–amino acid (aa) overlap, covering the complete sequence of the pp65 protein of CMV (UniProt ID: P06725). and a pool of 15‐mer peptides with 11–amino acid overlap, covering the complete sequence of the CMV IE‐1 protein (UniProt ID: P13202) were used. Briefly, 500 μL of fresh heparinised blood diluted in equal percentages with sodium chloride (NaCl) 0.9% were incubated at 37°C and 5% CO2, after stimulation with a pool of pp65 and IE‐1 CMV peptide libraries at a final concentration of 1 μg/mL and in the presence of costimulatory antibody CD28. For each sample a negative and positive PHA at 5 μg/mL control were included [36]. At incubation start, Brefeldin A at a final concentration of 5 μg/mL was added to culture medium. After 18 h, supernatants were removed and 100 μL of the remaining stimulated diluted blood was transferred to a new polystyrene tube and stained with an appropriate mix of conjugated antibodies including for the surface staining: Pacific, Blue‐conjugated anti‐CD45, APC‐conjugated anti‐CD8 and APC‐Cy7‐conjugated anti‐CD4, and subsequently incubated in darkness at 4°C for 20 min [37]. Then, red blood cells were lysed using the lysing solution (BD Biosciences, Franklin Lakes, NJ, USA), in darkness at room temperature for 20 min. Fix/Perm solution (BioLegend, San Diego, USA) was added prior intracellular staining performed with FITC‐conjugated anti‐IFNγ, PerCp‐Cy5.5‐conjugated anti‐TNFα and PE‐Cy7‐conjugated anti‐IL2, according to the manufacturer's instructions [37]. Samples were washed in Perm wash solution (BioLegend, San Diego, USA) according to the manufacturer's instructions. All the antibodies were purchased from BioLegend (Biolegend, San Diego, USA). Finally, samples were fixed in PBS containing 0.5% formaldehyde (Sigma‐Aldrich, St. Louis, USA) and samples were acquired using the MACSQuant Flow Cytometer (Miltenyi Biotec, Bergisch Gladbach, Germany) and analysed using FlowJo v10.8.1 software.

As previously described by Zingaropoli et al. [36] the cytokine background from the unstimulated condition was subtracted from the stimulated ones. Using the Boolean gate, all possible combinations of intracellular expression of IFNγ, IL2 and TNFα in cytokine‐producing T‐cells were evaluated. CMV‐specific T‐cell responses were assessed only in the PWH‐LA group, as the study was primarily designed to investigate longitudinal immunological changes after switching to long‐acting (LA) therapy. Consequently, these analyses were not performed in the CG.

### 2.5 Ethics Statement

The study IMMUNO‐ARV was approved by the LAZIO 1 local Ethics Committee (Study number: 57.23, protocol number ID 0068623/2023) once it was established that the research would be conducted in compliance with the indications of ethics and health protection, as established by the Ministry of Health of the Italian Government. Each subject gave written informed consent for participation in the study.

### 2.6 Statistical Analysis

Descriptive statistics were used to summarise the demographic and clinical characteristics of the enrolled PWH. Unless otherwise indicated, all data are given as median (25th and 75th percentiles). The nonparametric comparative Mann–Whitney test and the nonparametric Kruskal–Wallis test with Dunn's posttest were used for comparing medians between groups. Categorical variables were analysed using Fisher's exact test for contingency tables, and corresponding *p* values are reported in Table 1. Spearman's test was performed to assess the correlation between continuous variables (Spearman's coefficient [*r*] and statistical significance [*p*] are reported in the graphics). Longitudinal evaluation was performed using the nonparametric Friedman test. Delta(Δ) values were calculated at the individual level as the difference between week 48 (T48) and baseline (T0) measurements (Δ = T48 − T0) and were used to evaluate the magnitude and direction of changes over time and between groups. Between‐group comparisons of Δ values were performed using nonparametric tests, as described above. Given the exploratory nature of the analyses and the relatively small sample size, no formal correction for multiple testing was applied; thus, findings should be interpreted as hypothesis‐generating. Two‐tailed *p* ≤ 0.05 was considered statistically significant. Statistical analyses were performed using GraphPad Prism v.10.6.1 e for macOS. Correlation and delta analyses were additionally carried out using StataNow 18.5 SE—Standard Edition (StataCorp LLC, College Station, TX, USA; Licence No. 401809364100, DSPMI—Sapienza University of Rome).

**TABLE 1:** Demographic and clinical characteristics of the study population.

	PWH‐LA (*n* = 37)	PWH‐CG (*n* = 9)	*p*
Demographic and clinical characteristics			
Female, *n* (%)	13 (34%)	3 (33%)	> 0.999
Age, median (IQR) years	48 (35–56)	51 (37–52)	0.646
Body weight (kg), median (IQR)	74 (65–85)	69 (67–88)	0.832
AST (Aspartate Aminotransferase) (U/L), median (IQR)	22 (19–27)	20 (17–25)	0.462
ALT (Alanine Aminotransferase) (U/L), median (IQR)	18 (15–32)	15 (13–26)	0.336
Creatinine (mg/dL), median (IQR)	0.93 (0.78–1.02)	0.85 (0.83–1.06)	0.972
B Lymphocytes (cell/μL), median (IQR)	203 (150–299)	195 (133–349)	0.582
Triglycerides (mg/dL), median (IQR)	93 (73–132)	79 (56–104)	0.166
HDL (high‐density lipoprotein) (mg/dL), median (IQR)	52 (44–64)	59 (55–72)	0.116
LDL (low‐density lipoprotein) (mg/dL), median (IQR)	122 (107–141)	114 (87–134)	0.334
Total cholesterol (mg/dL), median (IQR)	199 (178–224)	197 (170–209)	0.385
NK (natural killer cells) (cell/μL), median (IQR)	283 (187–433)	232 (187–389)	0.511
HIV‐related parameters			
Time from HIV diagnosis, median (IQR) years	14 (7–23)	10 (4–18)	0.283
CD4 nadir cell count (cells/μL), median (IQR)	246 (89–408)	226 (104–310)	0.413
Current CD4 cell count (cells/μL), median (IQR)	705 (500–963)	644 (523–858)	0.752
Current CD8 cell count (cells/μL), median (IQR)	664 (490–936)	589 (379–834)	0.389
CD4/CD8 ratio, median (IQR)	0.97 (0.69–1.60)	1.42 (0.80–1.60)	0.331
HIV‐RNA < 50cp/mL (in PWH on HAART), *n* (%)	100%	100%	—
Co‐infection			
HBcAb positivity (%)	—	—	—
HCV Ab positivity (%)	—	—	
Anti‐CMV IgG positivity *n* (%)	34 (92%)	9 (100%)	> 0.999

ARV classes *n* (%)	Baseline	Current	
INSTI/NRTI dual	9 (24%)	2 (22%)	> 0.999
NNRTI/2 NRTI	10 (27%)	—	0.172
INSTI/NNRTI dual	4 (13%)	3 (33%)	0.123
INSTI/2 NRTI/	10 (27%)	3 (33%)	0.697
PI‐c/2 NRTI	3 (7%)	—	> 0.999
PI/INSTI/NNRTI	1 (2%)	—	> 0.999
PI‐c/INSTI	—	1 (12%)	0.0195

*Note:* Data are shown as median (interquartile range, IQR). *p* values were calculated using the Mann–Whitney U test for continuous variables and Fisher's exact test for categorical variables, as appropriate.

Abbreviations: CG, control group; *n*, number; PWH, people living with HIV.

## 3 Results

### 3.1 Demographic and Clinical Characteristics of PWH

Thirty‐seven PWH switching to CAB/RPV‐LA and nine continuing oral ART (PWH‐CG) were included in the study. Baseline demographic and clinical characteristics were comparable between groups (Table 1). Median age, sex distribution, body weight, liver and renal function, lipid profile and lymphocyte subsets did not differ significantly. Overall, both groups were well balanced at baseline.

### 3.2 Immunovirological Parameters

All participants maintained undetectable HIV‐RNA levels throughout follow‐up. Baseline immunological and clinical characteristics are summarised in Table 1. The duration of HIV infection tended to be longer in the PWH‐LA compared with PWH‐CG, while CD4 and CD8 T‐cell counts were slightly higher, resulting in a modestly lower CD4/CD8 ratio. In addition, all PWH tested negative for CMV DNA; however, more than 90% had detectable levels of IgG anti‐CMV. Participants had been previously exposed to multiple ART classes; NNRTI‐ and INSTI‐based combinations were the most frequent, with a similar distribution across groups. Overall, baseline characteristics were well balanced, with only minor, nonsignificant trends observed.

### 3.3 Assessing the Impact of CAB/RPV‐LA on Immune‐Activation and ‐Senescence in PWH

**FIGURE 1:**
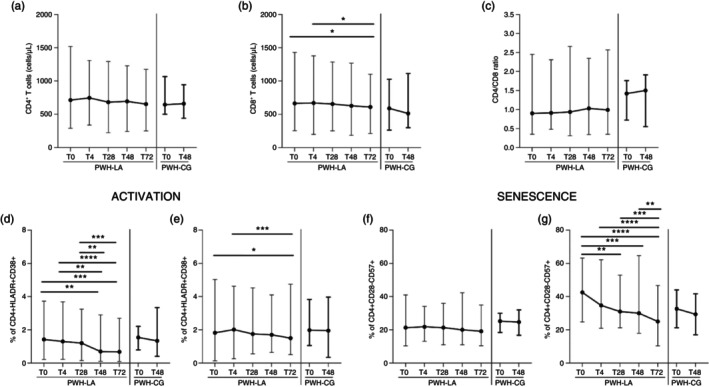
Absolute values (cells/μL) of CD4 (a), CD8 (b), CD4/CD8 ratio (c), of CD4 and CD8 immune‐activation T‐cells (d and f) and of CD4 and CD8 immune‐senescence T‐cells (f and g) measured over time in people with HIV switching to CAB/RPV long‐acting (PWH‐LA) and in the control group (PWH‐CG). PWH‐LA: people with HIV switching to CAB/RPV‐LA; PWH‐CG: people with HIV who remained on oral antiretroviral therapy; T0: Before the first CAB/RPV injection; T4: 4 weeks after the first injection; T28: 28 weeks after the first injection; T48: 48 weeks after the first injection; T72: 72 weeks after the first injection. Longitudinal changes in the PWH‐LA group were evaluated using the nonparametric Friedman test, while comparisons between T0 and T48 within the GC group were performed with the Wilcoxon matched‐pairs test. Differences across all time points and between groups were analysed using the Kruskal–Wallis test followed by Dunn's post hoc correction. Data are shown as median (horizontal lines). Statistical significance (*p*) is reported in the graphics, ****p < 0.0001; ***p < 0.001; **p < 0.01; *p < 0.05.

**FIGURE 2:**
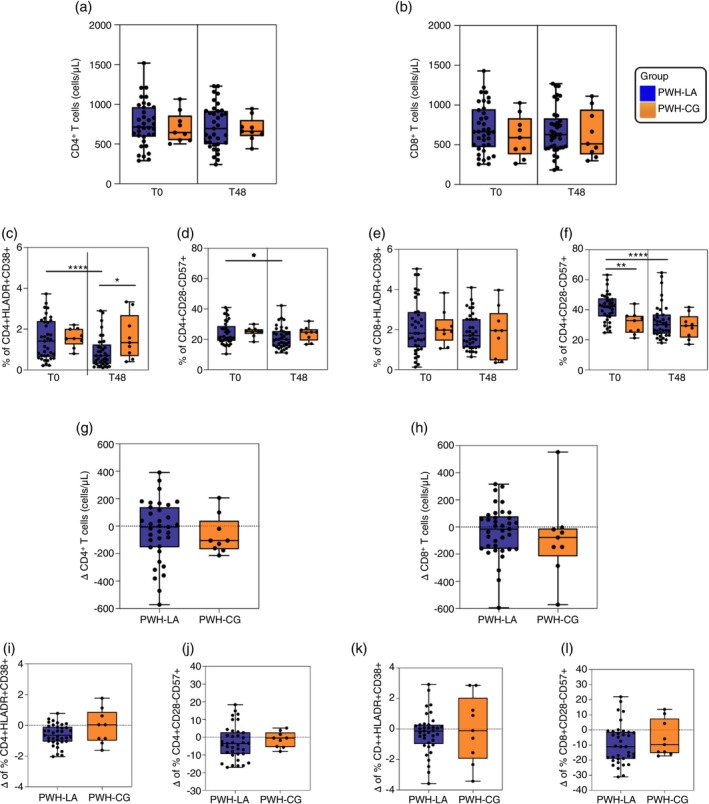
Absolute count of CD4 (a) and CD8 (b) T‐cells, immune‐activation and ‐senescence of CD4 (c and d), CD8 (e and f) T‐cell at baseline (T0) and Week 48 (T48) in people with HIV switching to CAB/RPV long‐acting (PWH‐LA) and in the control group (PWH‐CG), changes (Δ) in absolute CD4 (g) and CD8 (h) T‐cell count and in immune‐activation and ‐senescence of CD4 (i and j) and CD8 (k and l) T‐cells between baseline (T0) and Week 48 (T48) in people with HIV switching to CAB/RPV long‐acting (PWH‐LA) and in the control group (PWH‐CG). PWH‐LA: people with HIV switching to CAB/RPV‐LA; PWH‐CG: people with HIV who remained on oral antiretroviral therapy; T0: Before the first CAB/RPV injection; T48: 48 weeks after the first injection. Comparisons between T0 and T48 within each group were performed using the Wilcoxon matched‐pairs test, while comparisons between PWH‐LA and PWH‐GC were assessed with the Mann–Whitney test. Flow cytometry data for CD4 or CD8 immune‐activation and ‐senescence T‐cells are expressed as percentages. Data are shown as median (horizontal lines). Statistical significance (*p*) is reported in the graphics: ****p < 0.0001; ***p < 0.001; **p < 0.01; *p < 0.05.

In PWH‐LA, total CD4 levels remained stable over time, while CD8 levels decreased significantly at T72 versus T0 and T4 (*p* = 0.0157 and *p* = 0.0138) (Figure 1a,b). In contrast in PWH‐CG, the CG showed nonsignificant trends toward increased CD4 and decreased CD8 counts. (Figure 1a,b). The CD4/CD8 ratio remained overall stable in PWH‐LA and showed a modest nonsignificant increase at T48 in PWH‐CG (Figure 1c). In the entire cohort, the percentages of activated CD4 declined significantly at weeks T48 and T72 versus earlier time points (T0 *p* = 0.0002; T4: *p* = 0.0011 and *p* < 0.0001; T28: *p* = 0.0042 and *p* = 0.0001), while the percentage of activated CD8 declined significantly by week T72 versus T0 and T4 (*p* = 0.0258 and *p* = 0.0010). The CD4 and CD8 senescence decreased over time in both groups, with a significant reduction in CD8 senescent T‐cells at T28, T48 and T72 in PWH‐LA (T0 vs. T28 *p* = 0.0072; T48 *p* = 0.0004 and T0, T4, T28 and T48 vs. T72: *p* < 0.0001; *p* < 0.0001; *p* = 0.0002 and *p* = 0.0042) (Figure 2). In PWH‐CG, CD8 senescent T‐cells showed a downward trend. All medians and [IQR] are reported in Table S1.

### 3.4 Comparison Between PWH‐LA and PWH‐CG at Baseline and Week 48

At baseline (T0), absolute CD4 or CD8 T‐cell counts were comparable between PWH‐LA and PWH‐CG in (Figure 2a,b). By 48 weeks (T48), CD4 T‐cell counts remained similar, while CD8 T‐cell levels tended to be lower in PWH‐CG, although not significantly. When evaluating immune‐activation, in PWH‐LA the percentages of CD4 activated T‐cell decreased significantly by T48 versus T0 (*p* < 0.0001) (Figure 2c), and the percentage of CD8 activated T‐cell remained stable in both groups. Similarly, senescent CD4 and CD8 T cells declined in both groups at T48 versus T0 with a more pronounced and significant reduction in CD8 senescent cells in PWH‐LA, reaching statistical significance for CD8 senescent T‐cell (CD4: *p* = 0.05 and CD8: *p* < 0.0001) (Figure 2d,f). Between‐group comparisons at Week 48 showed lower levels of CD4 and CD8 activation in the PWH‐LA group compared with PWH‐CG, reaching statistical significance for CD4 activated T‐cells at T48 (*p* = 0.0263) (Figure 2c). No significant differences were observed between PWH‐LA and PWH‐CG in CD4 and CD8 senescent T‐cells, except for a significantly higher proportion of CD8 senescent T‐cells at baseline in PWH‐LA compared with the PWH‐CG (*p* = 0.0027). Analysis of individual changes (Δ = T48 − T0) confirmed these trends: PWH‐LA exhibited larger decreases in both activated and senescent T‐cells, particularly in CD4 activated and CD8 senescent T‐cells, compared with the PWH‐CG (Figure 2).

### 3.5 Humoral and T‐Cell Responses to CMV in PWH‐LA

**FIGURE 3:**
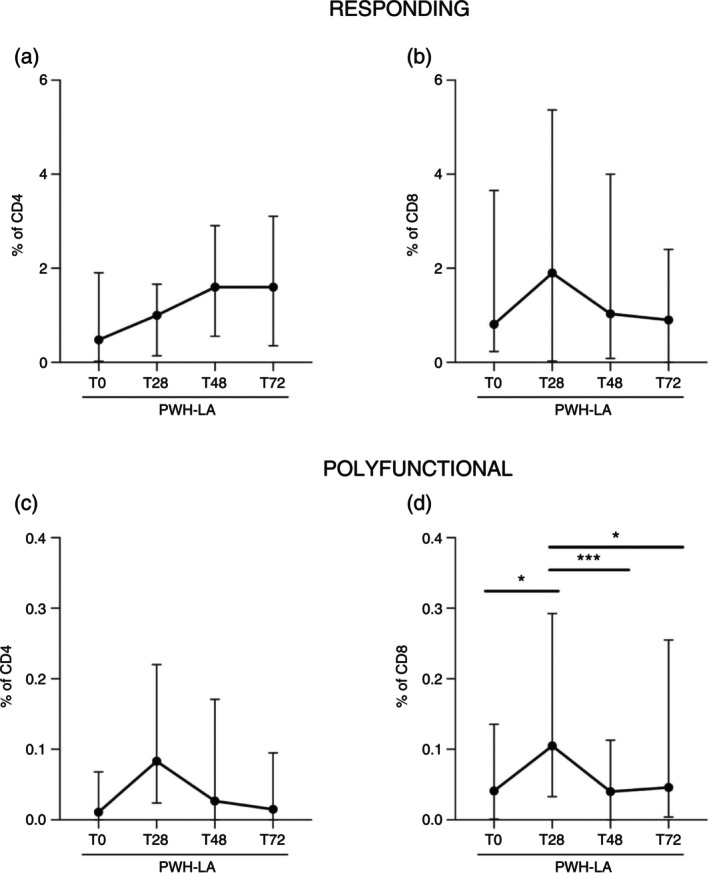
Percentage of responding T‐cell (a and b) and percentage of polyfunctional T‐cell (c and d) in PWH‐LA; T0: Before first injection of CAB/RPV; T28: 28 weeks after the first injection; T48: 48 weeks after the first injection; T72: 72 weeks after the first injection. Longitudinal changes in the PWH‐LA group were evaluated using the nonparametric Friedman test. Data are shown as median (horizontal lines). Statistical significance (*p*) is reported in the graphics: ****p < 0.0001; ***p < 0.001; **p < 0.01; *p < 0.05.

The analysis of CMV‐specific humoral and T‐responses was performed in 37 PWH‐LA at baseline (T0) and at 28 (T28), 48 (T48) and 72 (T72) weeks after switching. Anti‐CMV antibody levels remained overall stable throughout follow‐up with a slight decrease over time (Table S2). Regarding T‐cell responses, CD4 CMV‐specific T‐cell responses progressively increased after switching, reaching and maintaining elevated and stable levels at T48 and T72 (Figure 3a). In contrast, CD8 CMV‐specific responses showed a transient, nonsignificant rise at Week 28, followed by a return to baseline values at T48 and T72 (Figure 2b). A similar pattern was observed for polyfunctional T‐cell. Specifically, CD4 polyfunctional T‐cell increased at T28 and remained stable levels thereafter (Figure 3c). CD8 polyfunctional T‐cell peaked significantly at T28 compared with baseline (*p* = 0.0474), before declining at T48 and T72 (*p* = 0.0008 and *p* = 0.0414), returning to initial levels (Figure 3d).

### 3.6 Stratified Analyses and Correlation of Immunological Parameters and CMV‐Specific T‐Cell Responses in PWH‐LA

Stratified analyses were performed to assess changes in immunological parameters, including ΔCD4, ΔCD8, ΔCD4/CD8 ratio, CD4 and CD8 activated and senescent T‐cells, as well as CMV‐specific T‐cell responses, according to ART regimen, type of preswitch therapy (dual vs. nondual) and sex.

When stratified by ART class, mean changes in CD4 and CD8 T‐cells count varied widely across regimens, without consistent patterns or significant differences. PWH receiving NRTI/NRTI/INSTI regimens exhibited the largest median increase in CD4 T‐cell count, whereas those on PI‐based combinations showed modest decreases; however, variability within groups was high. No significant differences were observed between dual and nondual ART regimens or between males and females in ΔCD4, ΔCD8, ΔCD4/CD8 ratio or activation and senescence markers.

To explore the relationships between immune cell subsets and functional responses, we performed correlation analyses among changes (Δ = T48 − T0) in T‐cell count, activation, senescence and CMV‐specific responses within the PWH‐LA group (Table 2). A strong positive correlation was observed between ΔCD4 and ΔCD8 T‐cell count (*r* = 0.64, *p* < 0.001) (Table 2). In contrast, ΔCD4 changes were inversely correlated with ΔCD4 senescent T‐cell (*r* = −0.43, *p* = 0.02) (Table 2). Similarly, ΔCD8 senescent T‐cell showed a significant negative correlation with ΔCD8 T‐polyfunctional responses (*r* = −0.41, *p* = 0.03) (Table 2). No significant correlation emerged between changes in activation markers and polyfunctionality for either T‐cell subsets.

**TABLE 2:** Correlation matrix of immunological changes (Δ = T48 − T0) in PWH‐LA.

Variables	(1)	(2)	(3)	(4)	(5)	(6)	(7)	(8)	(9)	(10)	(11)
(1) delta_CD4	1.000										
(2) delta_CD8	0.642^*^	1.000									
	(0.001)										
(3) delta_ratio	0.236	−0.194	1.000								
	(0.186)	(0.273)									
(4) delta_CD4act	0.072	0.058	−0.214	1.000							
	(0.681)	(0.732)	(0.224)								
(5) delta_CD4senesc	−0.439^*^	0.150	0.374^*^	−0.183	1.000						
	(0.025)	(0.374)	(0.029)	(0.278)							
(6) delta_CD8act	0.167	0.247	0.152	0.291	−0.016	1.000					
	(0.337)	(0.141)	(0.390)	(0.081)	(0.925)						
(7) delta_CD8senesc	0.037	0.313	−0.087	0.080	0.026	−0.046	1.000				
	(0.834)	(0.059)	(0.627)	(0.638)	(0.880)	(0.788)					
(8) delta_CD4resp	0.081	0.079	0.096	−0.438^*^	0.010	−0.099	−0.260	1.000			
	(0.643)	(0.642)	(0.589)	(0.007)	(0.951)	(0.561)	(0.120)				
(9) delta_CD4poly	0.007	0.096	−0.057	−0.074	0.235	−0.071	−0.088	0.147	1.000		
	(0.966)	(0.572)	(0.749)	(0.665)	(0.161)	(0.675)	(0.605)	(0.386)			
(10) delta_CD8resp	−0.070	0.102	−0.096	−0.242	−0.034	0.035	−0.087	0.592^*^	0.094	1.000	
	(0.688)	(0.547)	(0.587)	(0.149)	(0.842)	(0.838)	(0.610)	(0.000)	(0.579)		
(11) delta_CD8poly	0.534^*^	0.232	0.250	−0.060	0.263	0.102	−0.412^*^	0.176	−0.040	−0.039	1.000
	(0.001)	(0.167)	(0.153)	(0.724)	(0.116)	(0.550)	(0.033)	(0.297)	(0.816)	(0.820)	

* *p* < 0.1.

** *p* < 0.05.

*** *p* < 0.01.

## 4 Discussion

Despite the remarkable effectiveness of ART in controlling HIV replication, daily oral regimens continue to pose adherence challenges, which can impact long‐term outcomes [17, 33, 38, 39]. Consequently, substantial efforts have focused on simplifying ART to improve adherence and PWH satisfaction. In this context, LA injectable regimens represent a major pharmacological innovation and a promising alternative to daily oral therapy [8, 9, 40].

In this study, we investigated the impact of CAB/RPV‐LA on immune‐activation and ‐senescence in PWH in stable immunovirological status. In addition, in a subgroup of individuals we explored the relationship between CMV‐specific immune responses and T‐cell phenotypic alterations, given the high prevalence of CMV co‐infection in PWH and its recognised role in chronic immune‐activation, immune‐senescence and non‐AIDS comorbidities [17, 18, 41, 42]. To our knowledge, this is the first study addressing these immunological parameters in CMV‐coinfected PWH switching to CAB/RPV‐LA.

Lymphoid immune‐activation during HIV infection has been well characterised in several studies in treatment naïve [43, 44] and ART experienced PWH [44, 45]. Following the switch to CAB/RPV‐LA we observed a significant reduction in activated CD4 and CD8 T‐cells over time. The decline in CD4 activation, a key driver of chronic inflammation in HIV [14, 46], suggests that LA therapy may enhance immune homeostasis [25, 26]. Similarly, the reduction in CD8 activation, which is associated with chronic inflammation, immune exhaustion and tissue damage in PWH [14, 46] supports the potential of LA regimens to mitigate chronic immune‐activation and its downstream complications [25, 26]. Regarding immune‐senescence, we observed a significant reduction in CD8 senescent T‐cell over 72 weeks, while the decline in CD4 senescent T‐cell was less pronounced. This differential effect between CD8 and CD4 T‐cell compartments likely reflects their distinct roles and dynamics in chronic HIV infection. CD8 T‐cells are more directly exposed to persistent antigenic stimulation and undergo clonal expansion and replicative senescence, particularly in the context of CMV co‐infection, a major driver of terminal differentiation and accumulation of CD28 − CD57+ T cells [41, 47]. In contrast, CD4 T‐cells exhibit a more heterogeneous and functionally diverse profile, which may result in a less pronounced and slower reversal of senescence‐associated features. The marked decline in CD8 senescence may therefore reflect the higher sensitivity of this compartment to changes in antigenic and inflammatory stimuli [47, 48, 49]. This is clinically relevant, given the association between immune senescence and age‐related comorbidities in PWH [14, 16, 41, 44].

In a subgroup analysis, we explored the relationship between CMV‐specific immune responses and T‐cell phenotypic changes. Anti‐CMV IgG levels remained stable and higher than in age‐ and sex‐matched healthy controls, consistent with persistent humoral immunity despite ART [50, 51, 52]. CMV‐specific T‐cell responses showed an early increase after the switch, particularly among CD4 and CD8 polyfunctional T‐cell subsets, followed by stabilisation or return to baseline levels in the absence of detectable CMV reactivation. This pattern suggests a transient immune reconfiguration, possibly reflecting early remodelling of the CMV‐specific T‐cell compartment after treatment switch.

Importantly, assessing CMV‐specific T‐cell responses requires consideration of both magnitude and functional quality. Polyfunctional T cells, characterised by the simultaneous production of multiple cytokines, are associated with more effective antiviral immunity [53, 54]. The observed increase in polyfunctional responses may therefore indicate improved functional capacity following the switch to LA therapy.

Notably, however, CMV‐specific polyfunctional T‐cell responses were positively correlated with senescent CD8 T‐cell subsets, highlighting the persistent impact of chronic CMV infection. Thus, despite evidence of qualitative improvements in T‐cell function, CMV likely continues to provide sustained antigenic stimulation that contributes to residual immune activation and senescence.

Overall, these findings support a model in which LA therapy contributes to an improved immunological profile, particularly through the reduction of immune activation and senescence, while persistent CMV infection continues to shape T‐cell dynamics and sustain a degree of residual immune activation even under optimal virological control.

Our study has several limitations. The duration of HIV infection tended to be longer in the PWH‐LA group than in the PWH‐CG group. This difference was not statistically significant, but it may still represent a potential confounding factor, as longer HIV duration has been associated with increased immune activation and senescence. Due to the relatively small sample size, particularly in the CG, adjusted analyses accounting for this variable could not be performed. Therefore, its potential impact on the observed immunological changes cannot be completely excluded, even though differences of this magnitude are generally considered more relevant during the early years of infection. CMV‐specific responses were assessed only in the LA group. Although baseline values provided an internal reference for longitudinal comparisons, the absence of evaluation of the CMV‐specific T‐cell response in the CG limits the ability to directly compare CMV‐related immune dynamics between treatment strategies and to attribute the observed changes specifically to the LA regimen. We did not evaluate HIV reservoirs or tissue drug penetration, factors that could further elucidate the mechanisms underlying the observed immunological changes. Finally, longer‐term follow‐up is needed to evaluate the durability of immunological effects observed with CAB/RPV‐LA.

## 5 Conclusions

In conclusion, (CAB/RPV)‐LA maintains viral suppression while contributing to reduce T‐cell activation and T‐cell senescence, supporting its potential to promote immune homeostasis in PWH. Nevertheless, persistent CMV co‐infection remains a key factor driving residual immune dysfunction. Future studies should further investigate the long‐term effects of LA ART on immune‐activation and ‐senescence in the context of CMV co‐infection to optimise treatment strategies and clinical outcomes in PWH.

## Author Contributions

M.L., A.C., C.D.B., R.M. conceived and designed the study. M.G. and E.T. drafted the manuscript. M.G., E.T., M.A.Z., F.D., S.V. performed experiments and analyses. L.A., F.M., A.G. collected samples and clinical data. M.G., E.T., F.M., F.D. participated in the experiments. A.C., M.A.Z., R.M., C.D.B., A.G. collected the data and organised the databases. M.G., E.T., M.A.Z., V.V., C.M.M., O.T., C.P., M.R.C. analysed and interpreted the data. M.L. and E.T. contributed to critical revision of the manuscript. All authors have read and approved the contents of the final manuscript.

## Funding

The authors have nothing to report.

## Conflicts of Interest

The authors declare no conflicts of interest.

## Supporting information


**Table S1:** Medians and IQR activated (CD38 + HLA‐DR+) and senescent (CD28‐CD57+) T‐cells.
**Table S2:** Medians and IQR of CMV specific immune responses in PWH‐LA.

## Data Availability

The data that support the findings of this study are available from the corresponding author upon reasonable request.
