# Supplementary material for: Long‐Acting Cabotegravir/Rilpivirine Reduces Immune‐Activation and ‐Senescence in People With HIV With CMV Co‐Infection
Source: Immunology. 2026 Jun 16;179(1):138–48. doi: 10.1111/imm.70154 (PMC13432221; doi:10.1111/imm.70154)
Supplement: Supplementary file 1 — Table S1: Medians and IQR activated (CD38 + HLA‐DR+) and senescent (CD28‐CD57+) T‐cells. Table S2: Medians and IQR of CMV specific immune responses in PWH‐LA. [file IMM-179-138-s001.docx]

**Supporting Information**

**Table S1.** Medians and IQR activated (CD38+HLA-DR+) and senescent (CD28-CD57+) T-cells.

|  | **PWH-LA** | | | | | **PWH-CG** | |
| --- | --- | --- | --- | --- | --- | --- | --- |
|  | T0 (n=37) | T4 (n=37) | T28 (n=37) | T48 (n=37) | T72 (n=37) | T0 (n=9) | T48 (n=9) |
| **CD4+HLA-DR+CD38+** | 1.42[0.64-2.39] | 1.30[0.56-2.08] | 1.20[0.61-2.01] | 0.70[0.30-1.25] | 0.68[0.20-1.10] | 1.54[1.25-2.01] | 1.34[0.69-2.68] |
| **CD8+HLA-DR+CD38+** | 1.82[1.11-2.88] | 2.01[1.38-3.21] | 1.75[1.34-2.50] | 1.70[1.13-2.50] | 1.50[1.10-2.10] | 1.98[1.45-2.51] | 1.95[0.47-2.82] |
| **CD4+CD28-CD57+** | 21.30[19.05-28.75] | 21.80[20.40-26.05] | 21.30[18.30-26.50] | 20.00[15.40-25.45] | 19.10[16.60-21.20] | 25.20[23.55-26.70] | 24.70[19.28-26.70] |
| **CD8+CD28-CD57+** | 42.50[35.55-47.60] | 34.70[26.62-42.25] | 31.0[25.78-39.20] | 30.0[24.12-36.85] | 25.0[29.20-32.70] | 32.70[24.65-36.0] | 29.40[21.52-35.70] |

PWH-LA: people with human immunodeficiency virus switching to cabotegravir-rilpivirine (CAB/RPV); PWH-CG: control group; n: number. T0: before first injection of CAB/RPV; T4: 4 weeks after the first injection; T28: 28 weeks after the first injection; T48: 48 weeks after the first injection; T72: 72 weeks after the first injection.

.

**Table S2**. Medians and IQR of CMV specific immune responses in PWH-LA.

|  | **PWH-LA** | | |  |  |
| --- | --- | --- | --- | --- | --- |
|  | T0 (n=37) | T28 (n=37) | T48 (n=37) | T72(n=37) |  |
| **CMV IgG levels (U/mL)** | 142[116.3-180] | 136[124.5-180] | 132[124-180] | 135[125-180] |  |
| **% of responding CD4** | 0.48[0.02-1.90] | 1.0[0.14-1.66] | 1.60[0.55-2.91] | 1.60[0.35-3.10] |  |
| **% of responding CD8** | 0.81[0.23-3.65] | 1.90[0.02-5.38] | 1.03[0.08-4.0] | 0.90[0.0-2.40] |  |
| **% of polyfunctional CD4** | 0.01[0.00-0.07] | 0.08[0.02-0.22] | 0.02[0.00-0.17] | 0.01[0.0-0.10] |  |
| **% of polyfunctional CD8** | 0.04[0.00-0.13] | 0.10[0.03-0.30] | 0.04[0.00-0.11] | 0.04[0.0-0.25] |  |

CMV: cytomegalovirus; IgG: immunoglobulin G;PWH-LA: people with human immunodeficiency virus switching to cabotegravir-rilpivirine CAB/RP. T0: before first injection of CAB/RPV; T28: 28 weeks after the first injection; T48: 48 weeks after the first injection; T72: 72 weeks after the first injection.
